# The Significance of Long Noncoding RNA H19 in Predicting Progression and Metastasis of Cancers: A Meta-Analysis

**DOI:** 10.1155/2016/5902678

**Published:** 2016-09-08

**Authors:** Wei Jing, Man Zhu, Xian-wei Zhang, Zhong-ya Pan, Shan-shan Gao, Hu Zhou, Shi-li Qiu, Chun-zi Liang, Jian-cheng Tu

**Affiliations:** ^1^Department of Laboratory Medicine, Clinical Laboratory Medicine and Center for Gene Diagnosis, Zhongnan Hospital of Wuhan University, Wuhan 430071, China; ^2^School of Laboratory Medicine, Hubei University of Traditional Chinese Medicine, Wuhan 430071, China

## Abstract

Recently, numerous studies indicate that H19 plays a key role in tumorigenesis, but the results have been disputed, especially in the aspects of tumor progression and metastasis. Therefore, we performed this meta-analysis to systematically summarize the relationship between H19 and cancers. We searched PubMed, the Cochrane Library, CNKI, and Chinese Wan Fang to identify eligible studies. Odds ratios and 95% confidence intervals were calculated to assess the effect size. A total of 13 studies were enrolled in this meta-analysis, which was performed by Revman5.3 and Stata11.0 software. Our meta-analysis showed that the expression of H19 was associated with distant metastasis in nongastrointestinal tumors (OR = 3.85, 95% CI = 1.31–11.36, *P* = 0.01) and, in gastrointestinal tumors (OR = 0.34, 95% CI = 0.15–0.78, *P* = 0.01), lymph node metastasis (OR = 2.04, 95% CI = 1.19–3.48, *P* = 0.009). Moreover, in gastric cancer, H19 expression was significantly related to histological grade (OR = 0.50, 95% CI = 0.29–0.86, *P* = 0.01), TNM stage (OR = 0.19, 95% CI = 0.11–0.33, *P* < 0.01), and tumor invasion depth (OR = 0.11, 95% CI = 0.04–0.27, *P* < 0.01). Therefore, H19 could serve as a potential marker for progression and metastasis evaluation of cancers.

## 1. Introduction

Cancers have the highest mortality in the world. In the year 2008 worldwide, 7.6 million people died of cancers; meanwhile 12.4 million new cases were suffering from cancers. It is forecasted that the deaths from cancers will increase to 13.1 million in 2030 [1]. The treatment of cancers can cause side effects in patients' physical and mental conditions, including pain, vomiting, fatigue, anepithymia, and body weight change [2]. Although patients can be successfully treated by surgery, chemotherapy, and interventional therapy, cancers may recur and be diagnosed in the advanced stage after the related symptoms appear and the 5-year survival rate is relatively low [3]. Unknowing the mechanisms of tumorigenesis is due to poor therapy and a high probability of relapse after treatment [4]. Recently, researchers focus on the biological markers associated with progression and metastasis in cancers. Therefore, identifying reliably diagnostic markers for cancers is urgently needed.

Noncoding RNAs are classified into small noncoding RNAs (miRNA) and long noncoding RNAs (lncRNAs) according to the transcript size. LncRNAs are defined as noncoding RNAs longer than 200 nucleotides with no protein-coding capacity [5]. Khachane and Harrison [6] demonstrated that the proportion of the lncRNA associated with cancer was 2 times higher than that of the protein-coding genes which have reference to cancer in the human genome. Increasing evidences have pointed to a relationship between lncRNAs and cancers, including metastasis, migration, and apoptosis, which change the original concept that lncRNA genes were just “noise” [7]. For example, urothelial carcinoma-associated 1 (UCA1) can directly bind to miR-216b, and the abnormal expression of UCA1 in HCC is correlated with tumor-node-metastasis (TNM) stage and metastasis [8]. In breast cancer, the overexpression of the plasmacytoma variant translocation 1 gene (PVT1) could inhibit the apoptosis of tumor cells, which is involved in the pathophysiology of breast cancer [9]. Recently, researches indicated that many lncRNAs played important roles in cancers.

LncRNA H19, which is the first discovered lncRNA by Brannan, is a paternally imprinted gene located close to the telomeric region of chromosome 11p15.5, which is frequently involved in tumors [10, 11]. Matouk et al. [12] indicated that H19 was not expressed in tissues after birth and reexpressed in the tumor tissues. H19 is associated with the tumorigenesis and invasion, partially via the regulation of carcinogenic miRNA-675 which locates in its first exon [13]. Barsyte-Lovejoy et al. [14] showed that c-myc can induce the expression of H19 and when acetylation occurred in its promoter region, transcription initiated. H19 was upregulated in several different tumors, including esophageal cancer, gastric cancer, and breast cancer [15–17]. Numbers of researches about H19 were done to explore the mechanism in cancers. Therefore, we conducted a meta-analysis to evaluate the value of H19 with tumor metastasis and progression in a larger sample size of patients.

## 2. Materials and Methods

### 2.1. Publication Search

To obtain relevant articles for this meta-analysis, we searched the databases PubMed, Cochrane library, CNKI, and Chinese Wan Fang for studies published up to July 2016. Both medical subject heading terms and free-text words were used in the databases to increase sensitivity. The following search keywords were used: “H19 and cancer,” “progression and metastasis,” “long noncoding RNA H19.” Meanwhile, we examined all articles in these eligible studies to identify additional relevant literature that had not been retrieved from the databases.

### 2.2. Inclusion and Exclusion Criteria

Eligible studies in this meta-analysis had to meet the following standards: patients in the study were diagnosed with cancers; researches were association between H19 and cancer; sufficient published data were provided to calculate odds ratios (ORs) and 95% confidence interval (95% CI). If there were duplicated data, we chose the most complete data or the most recent one. Exclusion criteria were as follows: studies without usable data, case reports, reviews, letters, and conference abstracts.

### 2.3. Data Extraction

Two investigators extracted relevant data from the eligible studies independently, including the first author, year of publication, country, tumor type, sample, sample size, and cut-off value.

### 2.4. Statistical Analysis

ORs and 95% CIs were used to assess the association between H19 and clinic features in cancers. The features included gender, lymph node status, and distant metastasis. Meanwhile, histological grade, TNM stage, and tumor invasion depth were extracted from the articles about gastric cancer. The clinicopathological factor of gender was divided into males and females. As for lymph node status (LNM) and distant metastasis (DM), we separated them into positive and negative. Similarly, histological grade included low-grade and high/middle-grade, respectively. According to the American Joint Committee on Cancer (AJCC) staging system [18], TNM stage was separated into two parts, which were early stage (≤II) and late stage (≥III). The tumor invasion depth was divided into T0-T1 and T2 or above. We used Revman5.3 software (Revman, the Cochrane Collaboration) to perform the meta-analysis and evaluate heterogeneity between studies by Cochrane *Q*-test and *P* values. If heterogeneity was present (*I* ^2^ ≥ 50% or *P* ≤ 0.05), random-effect model was used to calculate pooled ORs. If not, the fixed-effect model was more appropriate [19, 20]. The Stata11.0 software (Stata, College Station) was performed to evaluate the sensitivity and publication bias of the studies. Publication bias was evaluated by Begg's test; *P* < 0.05 was considered statistically significant.

## 3. Results

### 3.1. Characteristics of Included Studies

**Figure 1:**
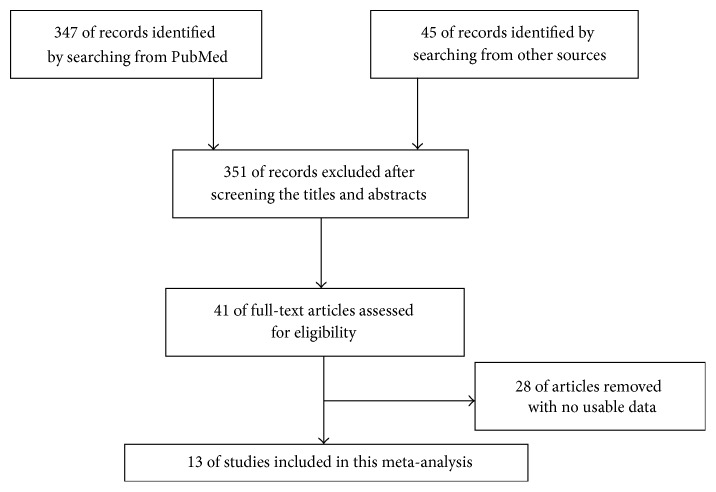
The flow diagram of this meta-analysis.

As shown in the flow diagram (Figure 1), we searched 392 articles in the databases. After screening the titles and abstracts, 351 of records were removed. Then because of no usable data, 28 papers were excluded. As a result, a total of 13 articles were included in the current meta-analysis [3, 11, 21–31].

Table 1 summarized the main characteristics of the included 13 studies ranging from 2005 to 2016. Among these 13 studies, 11 were from China, 1 from Japan, and 1 from Brazil. Specimens were divided into 8 types: 4 gastric cancer (GC) [3, 22, 24, 27]; 1 non-small-cell lung cancer (NSCLC) [21], 1 renal cell carcinoma (RCC) [11], 2 gallbladder cancer (GBC) [23, 30], 1 head-and-neck squamous cell carcinomas (HNSCC) [25], 2 esophageal cancer (EC) [26, 29], 1 ovarian cancer (OC) [28], and 1 colorectal cancer (CRC) [31]. Sample types included tissues and plasma. 13 studies enrolling 872 participants were with a maximum sample size of 133 and a minimum sample size of 20 patients. Because of variations in the cut-off definitions, the cut-off values were different in these studies.

### 3.2. Association between H19 and Clinicopathological Parameters

**Figure 2:**
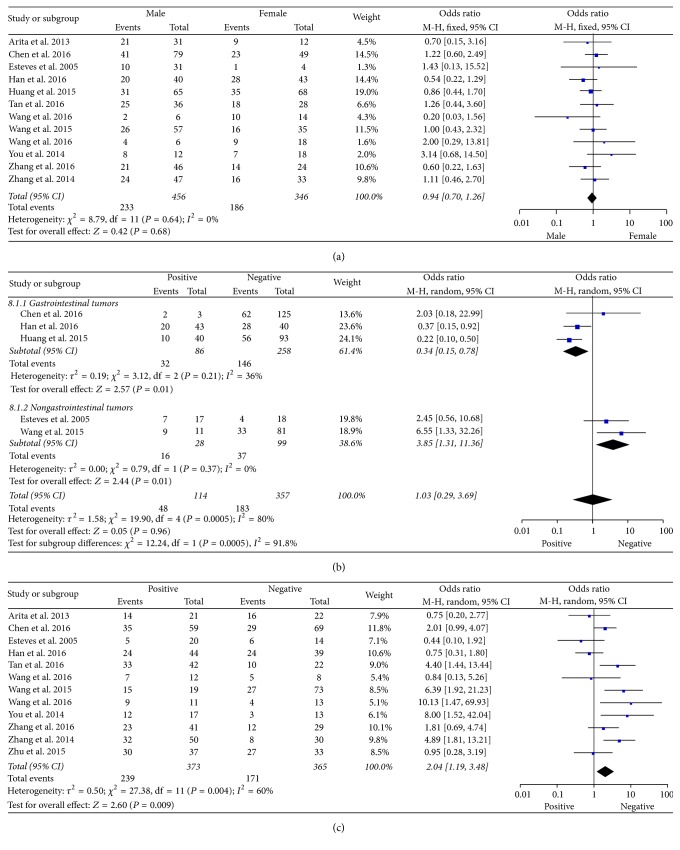
Forest plot for the association between H19 expression levels with clinical parameters in cancers. (a) Gender. (b) Distant metastasis. (c) Lymph node metastasis.

A total of 802 patients enrolled in 12 studies reported that the expression levels of H19 were related to gender. Analysis showed that the expression levels of H19 were not associated with the gender of patients (OR = 0.94, 95% CI = 0.70–1.26, *P* = 0.68, fixed-effect) (Figure 2(a)). Five studies showed the relationship between H19 and distant metastasis. Due to the different types of cancers, we divided cancers into gastrointestinal tumors and nongastrointestinal tumors. Then we performed subgroup analysis. Result by cancer type indicated that H19 expression was significantly related to distant metastasis in nongastrointestinal tumors (OR = 3.85, 95% CI = 1.31–11.36, *P* = 0.01, random-effect) and also in the gastrointestinal tumors (OR = 0.34, 95% CI = 0.15–0.78, *P* = 0.01, random-effect). However, we found that there was no correlation between H19 and these two types of cancers (nongastrointestinal and gastrointestinal cancers) (OR = 1.03, 95% CI = 0.29–3.69, *P* = 0.96, random-effect) (Figure 2(b)). Finally, we analyzed the association between H19 and lymph node metastasis, and the result elaborated that lymph node metastasis occurred with the expression levels of H19 (OR = 2.04, 95% CI = 1.19–3.48, *P* = 0.009, random-effect) (Figure 2(c)).

### 3.3. Association between H19 and Clinicopathological Parameters in GC

**Figure 3:**
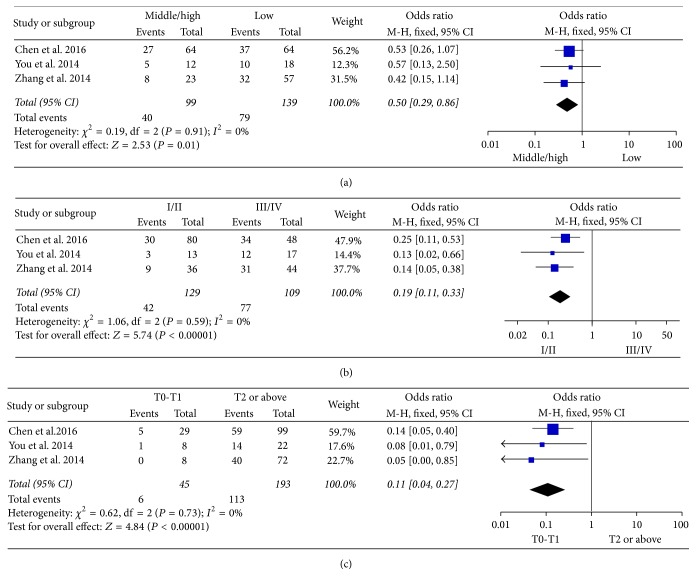
Forest plot for the association between H19 expression levels with clinical parameters in GC. (a) Histological grade in GC. (b) TNM in GC. (c) Tumor invasion depth in GC.

Four of the 13 studies enrolling 281 patients indicated that H19 expression was related to the clinicopathological characteristics in GC [3, 22, 24, 27]. In those studies, H19 expression was significantly associated with histological grade (OR = 0.50, 95% CI = 0.29–0.86, *P* = 0.01, fixed-effect), TNM stage (OR = 0.19, 95% CI = 0.11–0.33, *P* < 0.00001, fixed-effect), and tumor invasion depth (OR = 0.11, 95% CI = 0.04–0.27, *P* < 0.00001, fixed-effect) (Figures 3(a)–3(c)) in GC.

### 3.4. Publication Bias and Sensitivity Analysis

**Figure 4:**
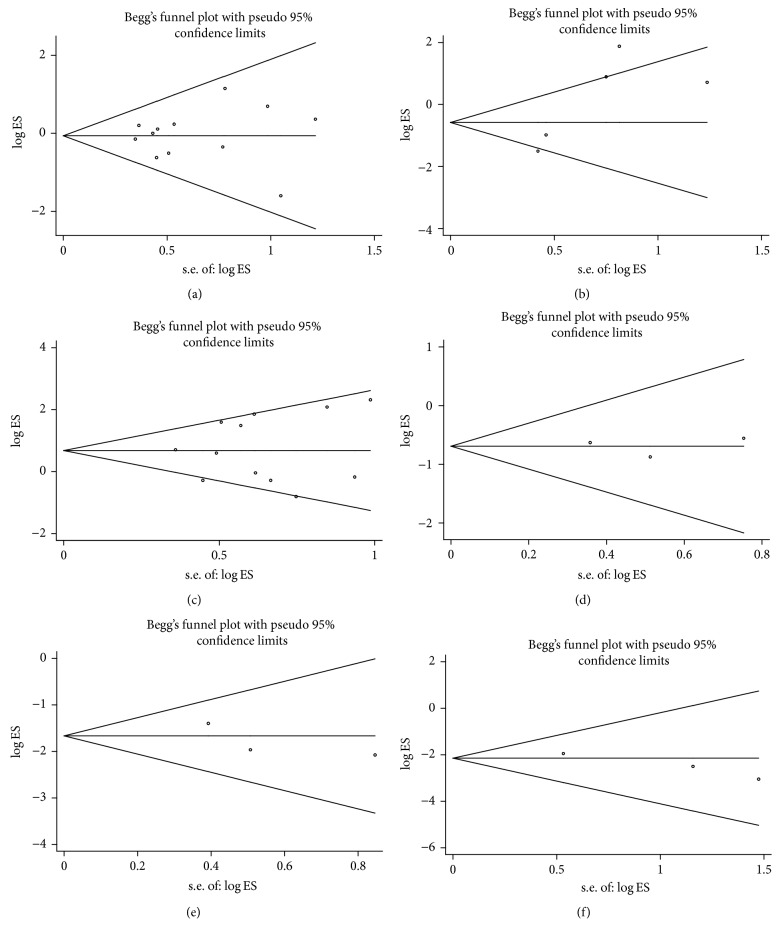
Begg's test for publication bias. (a) Gender. (b) Distant metastasis. (c) Lymph node metastasis. (d) Histological grade in GC. (e) TNM in GC. (f) Tumor invasion depth in GC.

**Figure 5:**
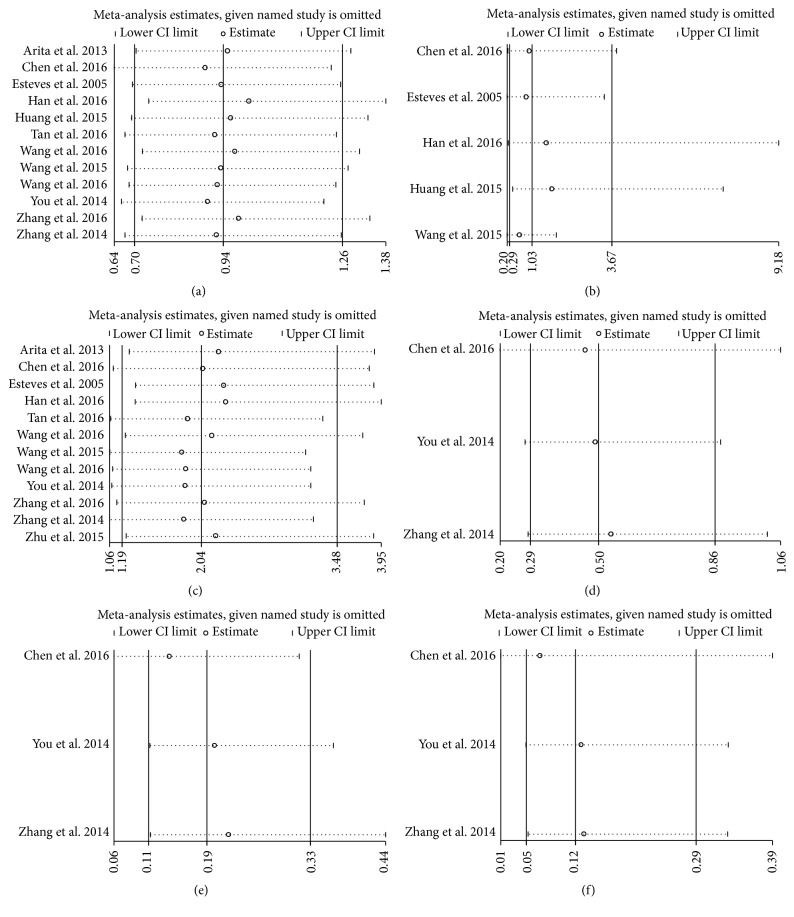
Sensitivity analyses of the studies. (a) Gender. (b) Distant metastasis. (c) Lymph node metastasis. (d) Histological grade in GC. (e) TNM in GC. (f) Tumor invasion depth in GC.

We used Begg's test to evaluate the publication bias, respectively (Figures 4(a)–4(f)). In our meta-analysis, Begg's test indicated that there was no publication bias in all subgroups and all the values of *P* > 0.05. Sensitivity analysis was performed by Stata11.0 software to assess whether the individual study affected the overall results. The results suggested that individual study had little influence on our final results (Figures 5(a)–5(f)), which demonstrated that our results were relatively stable and credible.

## 4. Discussion

The occurrence of cancers is a multifactor, multistep, and complex process. Due to the lack of early prediction index, numerous patients were diagnosed in their late stage. In recent years, lncRNA plays increasingly important roles in epigenetics, pretranscription, and posttranscription, which is also becoming a research hotspot [32]. However, only a few diverse hypothetical mechanisms were presented by which the lncRNAs could exert their effects: interfering in the expression of the adjacent encoding protein gene [33]; participating in transcription and chromatin-modifying and DNA methyltransferases to specific genomic [34]; binding with functional protein [35]; being the precursors of miRNAs and affecting targets gene of miRNA [36, 37]; regulating signaling pathway via combining with chromosome [38, 39].

H19 is one of the cancer-related lncRNAs, which has an oncogenic function and is strongly expressed in cancers, such as colorectal cancer, osteosarcoma, and bladder cancer [40–42]. In 2013, Luo et al. [43] demonstrated that H19 was remarkably increased in bladder cancer tissues, comparing with adjacent normal control tissues, and promoted bladder cancer cells proliferation in vitro. Vennin et al. [44] elaborated that H19/miR-675 enhanced the cell proliferation and migration in vitro and increased tumor growth and metastasis in vivo. Meanwhile, H19 decreased ubiquitin ligase E3 family (c-Cbl and Cbl-b) which suppressed tumorigenesis through its microRNA. Thus, H19 could be considered as a potential prognostic factor for various cancers.

The important reason for the low 5-year survival rate is the occurrence of distant metastasis and lymph node metastasis. Recently, researchers indicated that H19 inhibited endogenous let-7 function, causing derepression of HMGA2 which could mediate epithelial-mesenchymal transition (EMT) in pancreatic ductal adenocarcinoma (PDAC), and contributed to PDAC metastasis [45]. Raveh et al. [46] indicated that H19 played an important role in EMT process, which showed that H19 exerted a metastatic function in cancers. P53 is known as a tumor suppressor gene and arrests tumor growth and metastasis [47]. In 2012, Yang et al. [48] found that H19 was associated with p53 and this association led to partial p53 inactivation. Also, in bladder cancer cells, H19 positively regulated miR-675 expression, which could inhibit p53 activation [49]. Therefore, H19 could be used to predict the occurrence of metastasis in cancers. In this meta-analysis, we found that high levels of H19 were more prone to lead to lymph node metastasis (OR = 2.04, 95% CI = 1.19–3.48, *P* = 0.009). Furthermore, the level of H19 expression in DM-positive group was 3.85-fold higher than in the DM-negative group in nongastrointestinal tumors (OR = 3.85, 95% CI = 1.31–11.36, *P* = 0.01). However, in gastrointestinal tumors, our result showed that the expression of H19 in DM-negative group was 2.94-fold higher than in the DM-positive group (OR = 0.34, 95% CI = 0.15–0.78, *P* = 0.01); insufficient sample size was the possible reason for the different results in various cancers. Therefore, further studies should be done with larger sample sizes.

In subgroups analysis, we found that the levels of H19 were significantly related to the histological grade (OR = 0.50, 95% CI = 0.29–0.86, *P* = 0.01), TNM stage (OR = 0.19, 95% CI = 0.11–0.33, *P* < 0.01), and tumor invasion depth (OR = 0.11, 95% CI = 0.04–0.27, *P* < 0.01) in GC. Previous study demonstrated that H19 was an important factor in GC tumorigenesis and metastasis by interacting with its target gene ISM1 that had a dual function in endothelial cell survival and cell death [16]. In included studies, Chen et al. [27] found that knockdown of H19 could inhibit GC cell migration and invasion partly via regulating E-cadherin protein expression which can decrease the occurrence of invasiveness. Therefore, H19 can serve as a sentinel, indicating the development of GC. However, the relationship between H19 expression and other cancers might need more studies to illuminate.

It should be stressed that there were limitations in our analysis. Most studies reported positive results, but those with negative results are generally less likely to be published. In addition, there were insufficient data to fully confirm the association between H19 and clinicopathological characteristics, which needs more studies. Finally, the limited number of studies may affect the results of subgroups.

## 5. Conclusions

In conclusion, this meta-analysis suggested that H19 might predict progression and metastasis in cancers. We firstly explored the correlation of H19 expression levels with lymph node metastasis and distant metastasis in cancers. Meanwhile, the expression of H19 was associated with histological grade, TNM, and tumor invasion depth in GC. Therefore, our study demonstrated that H19 might be a predictive factor for assessing progression and metastasis in cancers.

## Figures and Tables

**Table 1:** Characteristics of studies included in this meta-analysis for H19.

Author	Year	Country	Tumor type	Sample	Sample size	Cut-off value
Zhang [3]	2014	China	GC	Tissue	80	Mean
Zhang [21]	2016	China	NSCLC	Tissue	70	Median
Arita [22]	2013	Japan	GC	Plasma	43	0.32
Wang [11]	2015	China	RCC	Tissue	92	3.8-fold change
Wang [23]	2016	China	GBC	Tissue	20	NA
You [24]	2014	China	GC	Tissue	30	Median
Esteves [25]	2005	Brazil	HNSCC	Tissue	35	Absence/presence
Huang [26]	2015	China	EC	Tissue	133	Median
Chen [27]	2016	China	GC	Tissue	128	Median
Zhu [28]	2015	China	OC	Tissue	70	NA
Tan [29]	2016	China	EC	Tissue	64	NA
Wang [30]	2016	China	GBC	Tissue	24	Median
Han [31]	2016	China	CRC	Tissue	83	3.8-fold change

GC: gastric cancer, NSCLC: non-small-cell lung cancer, RCC: renal cell carcinoma, GBC: gallbladder cancer, HNSCC: head-and-neck squamous cell carcinomas, EC: esophageal cancer, OC: ovarian cancer, and CRC: colorectal cancer.
